# Intestinal malrotation combined with superior mesenteric artery syndrome: a case report

**DOI:** 10.3389/fped.2026.1775767

**Published:** 2026-04-01

**Authors:** Ning An, Liangfu Jiang, Xingyun Mao, Guangping Zhang, Guoxiu Han, Hui Li, Rongpeng Zhang, Yiming Wang, Yu Liu

**Affiliations:** Department of Pediatrics Surgery, Linyi People’s Hospital, Affiliated Hospital of Shandong Second Medical University, Linyi, Shandong, China

**Keywords:** case report, children, intestinal malrotation, Ladd's operation, superior mesenteric artery syndrome

## Abstract

**Background:**

Intestinal malrotation (IM) is a developmental anomaly of the intestine characterized by abnormal rotation of the midgut around the superior mesenteric artery (SMA) axis. This condition is more prevalent in children under 1 year of age. Superior mesenteric artery syndrome (SMAS) is a rare disorder caused by a decreased angle between the abdominal aorta (AA) and the SMA, leading to compression of the horizontal segment of the duodenum. Both conditions present with symptoms such as abdominal pain and vomiting. A preoperative diagnosis that relies exclusively on clinical manifestations and imaging examinations may lead to misdiagnosis or overlooking of the condition.

**Case description:**

A 13-year-old female patient was admitted to the hospital with a 12-h history of abdominal pain accompanied by vomiting and reduced passage of flatus. Physical examination revealed tenderness in the upper abdomen and periumbilical region, along with diminished bowel sounds. Preoperative imaging revealed a “vortex sign” and a reduced angle between AA and SMA (10°). During surgery, a 270° reverse rotation of the intestine was observed. The angle between the AA and SMA was further reduced, and SMA was positioned in close proximity to the duodenum. Obstruction of the distal descending duodenum was also observed. The patient was diagnosed with reverse intestinal malrotation (RIM) combined with SMAS. The patient underwent Ladd's procedure and duodenojejunostomy and recovered completely after the operation. No abnormalities were observed during the follow-up.

**Conclusions:**

In patients presenting with IM accompanied by abdominal pain and vomiting, clinicians should consider the possibility of RIM and SMAS. A comprehensive preoperative evaluation is essential to exclude complex gastrointestinal malformations, thereby optimizing the surgical strategy and enhancing postoperative recovery.

## 1 Introduction

Intestinal malrotation (IM) is a developmental abnormality of the intestine caused by disruption of the normal rotational movement of the midgut around the superior mesenteric artery (SMA) during embryonic development (1). It primarily occurs during the neonatal period, and its incidence decreases significantly after the age of one year (2). Although IM is relatively common, intestinal volvulus may occur if not treated promptly, which can rapidly progress to intestinal necrosis and potentially lead to death (3). Therefore, timely surgical intervention, such as Ladd's procedure, is the cornerstone of IM treatment. While IM is common in clinical practice, reverse intestinal malrotation (RIM) is a rare subtype of IM. In RIM, the intestine rotates clockwise around the SMA, which is located anterior to the transverse colon and may cause incomplete obstruction of the transverse colon due to compression (4).

Superior mesenteric artery syndrome (SMAS) is another rare intestinal obstruction, characterized by compression of the horizontal segment of the duodenum by the SMA or its branches. The clinical manifestations of this condition are often non-specific, leading to frequent misdiagnoses. Patients typically present with symptoms such as chronic abdominal pain and weight loss. Prompt and accurate diagnosis is essential for implementing appropriate conservative treatment strategies or timely surgical interventions, thereby mitigating the risk of severe complications (5, 6).

In this study, we report a rare case of a patient with RIM and SMAS. This combination is infrequently observed and is thought to be associated with embryonic developmental abnormalities underlying the pathogenesis of these two anatomical variations. Preoperative imaging revealed the presence of a “vortex sign” and a reduction in the angle between the abdominal aorta (AA) and SMA. Laparoscopic exploration confirmed a 270° reverse intestinal rotation, decreased angle between AA and SMA, and SMA positioned in close proximity to the duodenum, resulting in distal duodenal obstruction. However, no significant obstruction or malformation was observed in the distal small intestine or colon. Given the complexity of the condition, the surgical approach was converted from laparoscopy to laparotomy. Ladd's procedure and duodenojejunostomy were performed. The patient demonstrated favorable recovery following the surgery. This case report was prepared in accordance with the CARE reporting checklist.

## 2 Case presentation

**Figure 1:**
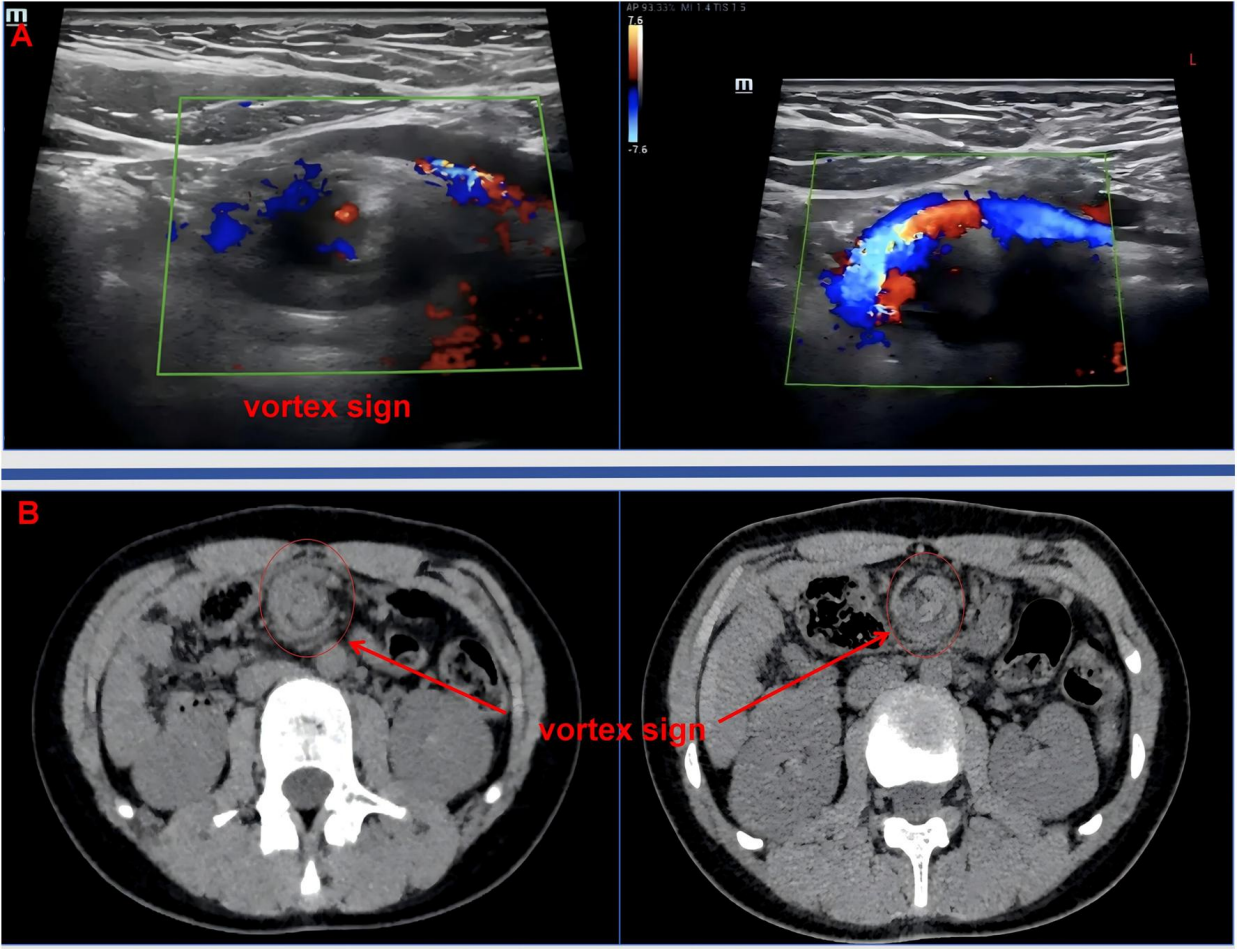
Preoperative imaging examinations. **(A)** US: SMA served as the center, with the mesentery, blood vessels, and intestines forming a vortex around it. Abnormal positioning of SMA and superior mesenteric vein was evident. **(B)** Classical “vortex sign.”

**Figure 2:**
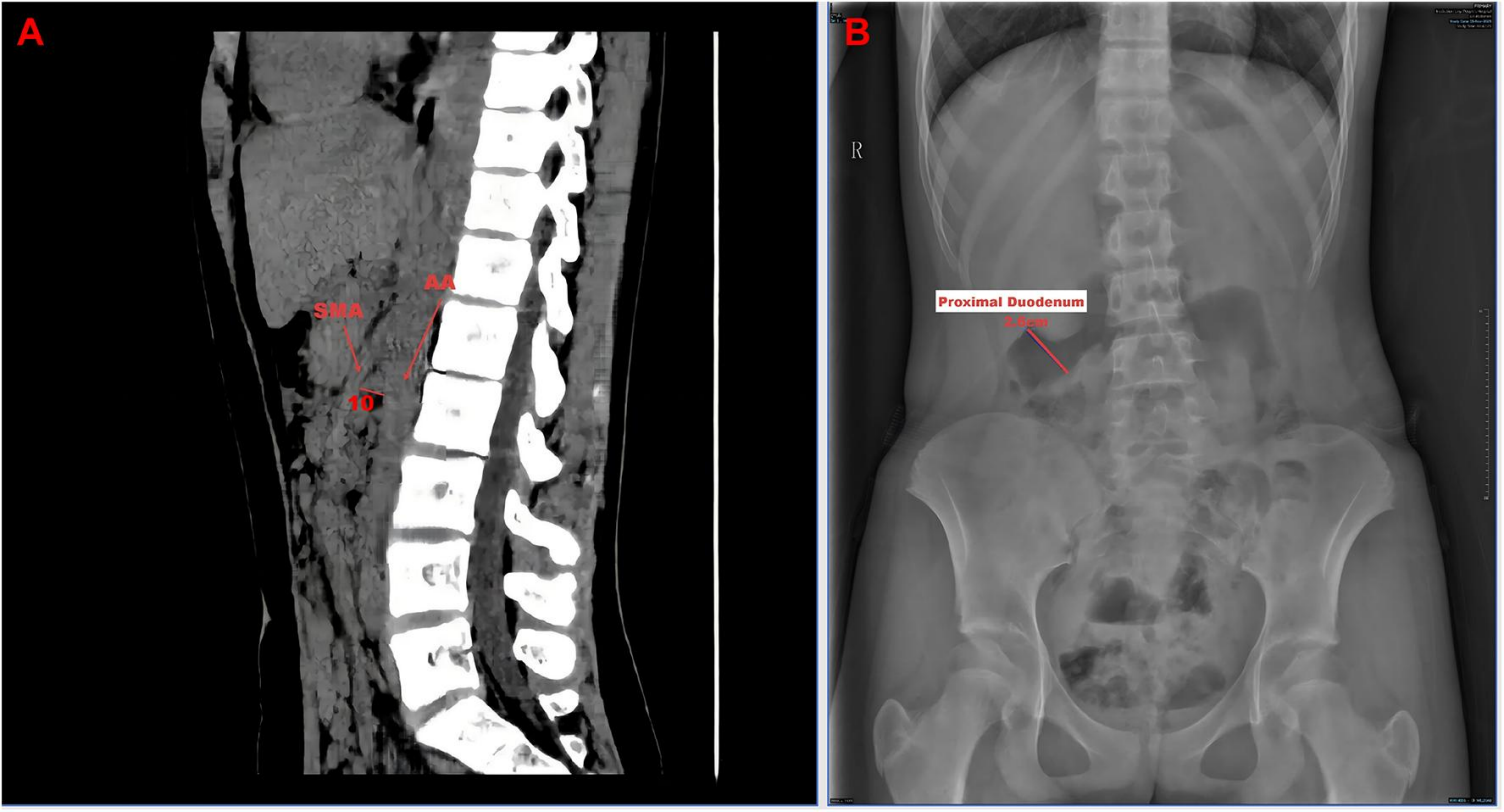
Preoperative imaging examinations. **(A)** CT scan depicting a decreased angle between SMA and AA. **(B)** X-ray demonstrating dilation of the proximal duodenum.

A 13-year-old female patient was admitted to Linyi People's Hospital in November 2025, presenting with a 12-h history of abdominal pain. The patient reported postprandial abdominal pain, predominantly localized to the umbilicus. The pain exhibited positional variability, intensifying when the patient was standing and alleviating in the prone position. Moreover, the patient experienced reduced passage of flatus, nausea, and non-bilious vomiting. Notably, there were no signs of fever, hematemesis, or melena. The patient's medical history, including perinatal and family histories, was unremarkable. Physical examination revealed the following findings: T: 36.2 °C; HR: 69 beats/min; BP: 114/72 mmHg; weight: 42 kg; height: 162 cm; and body mass index (BMI): 16 kg/m^2^ (II° malnutrition). The Screening Tool for the Assessment of Malnutrition in Pediatrics (STAMP) score was 2 (disease risk 2 + nutritional intake 0 + growth status 0; moderate risk of malnutrition) (7). Abdominal examination revealed a normal contour, with no evidence of gastrointestinal distension or visible peristaltic waves. No varicosities were observed on the abdominal wall. The abdomen was soft, with pronounced tenderness in the upper abdomen and periumbilical region. Bowel sounds were significantly reduced. Abdominal ultrasound (US) and computed tomography (CT) scans revealed findings consistent with the “vortex sign,” suggestive of mesenteric torsion (Figures 1A,B, Supplementary Material 1). In addition, abdominal CT revealed a reduction in the angle (10°) between the AA and SMA (Figure 2A), whereas plain abdominal radiography demonstrated proximal duodenal dilation (Figure 2B).

**Figure 3:**
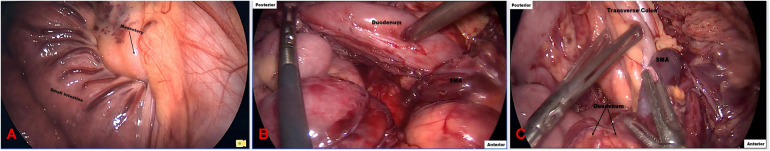
Intraoperative findings. **(A)** The duodenal blood supply was slightly compromised, but no necrosis was observed. **(B)** SMA compressing a portion of the duodenum. **(C)** Anatomical relationship among the mesenteric vessels, duodenum, and jejunum, depicting SMA positioned anterior to the duodenum and transverse colon.

The surgical procedures included conversion from laparoscopic to open surgery, reduction of intestinal torsion, Ladd's procedure, and duodenojejunostomy. Laparoscopic examination revealed a 270° clockwise torsion of the small intestine around the SMA, with slightly compromised blood supply, but no evidence of necrosis (Figure 3A). The small intestine was untwisted in a counterclockwise direction until normal positioning was achieved. Ladd's bands were found compressing the duodenum, resulting in distal obstruction of the descending segment. All adhesion bands involving the duodenum and upper jejunum were dissected, and the cecum was mobilized. Following the release of Ladd's bands, the SMA was positioned anterior to the duodenum and transverse colon, exerting pressure on a portion of the duodenum (Figure 3B). The interrelationship between the mesenteric vessels, colon, and duodenum is depicted in Figure 3C (Supplementary Material 2). A side-to-side anastomosis between the duodenum and jejunum was performed to bypass the area of compression. Intraoperative assessment revealed that the distal small intestine and colon were patent, with no significant obstruction or intestinal malformation. An appendectomy was performed, and the intestinal tract was appropriately repositioned. The surgical procedure lasted 4 h, with an estimated intraoperative blood loss of 200 mL.

On the 5th postoperative day, the patient passed flatus, and bowel movements resumed on the 6th day. The abdominal/pelvic drain yielded hemoserous discharge with an initial output of 450 and 400 mL on postoperative day 1, respectively, but output decreased to 35 mL of clear secretions by postoperative days 9 and 10. In view of this, the drain was removed. Throughout this period, the patient's blood electrolyte and albumin levels remained within the normal range, and daily urine output was normal. The patient remained alert and in good spirits, with no signs of irritability or malaise. Skin turgor was normal, and there were no reports of headache, dizziness, nausea, palpitations, dehydration, electrolyte imbalance, or hypoproteinemia. On the 8th postoperative day, the patient initiated a liquid diet, which was gradually advanced to a normal diet. She had a good appetite, with no episodes of nausea or vomiting, and normal bowel movements. The patient was discharged on the 16th postoperative day in satisfactory condition.

Based on the clinical manifestations and intraoperative findings, the final diagnoses were as follows: (1) RIM; (2) SMAS; (3) intestinal torsion; and (4) intestinal obstruction. Postdischarge follow-up assessments were conducted at 1, 2 weeks, and 1 month. During follow-up, the patient reported no abdominal pain, demonstrated good wound healing, maintained a healthy appetite, had normal bowel movements, and exhibited no abnormalities on abdominal ultrasound. One month postoperatively, her body weight was 43.8 kg, reflecting an increase of 1.8 kg compared to preoperative weight. The timeline of the patient's treatment is summarized in Table 1.

**Table 1:** Timeline of treatment procedure for the patient.

Time	Clinical feature	Treatment
15 November 2025	A 13-year-old girl was admitted to the emergency department with abdominal pain for 12 h. Preoperative examination suggested IM.	During emergency laparoscopic exploration, the small intestine was found to have a 270° clockwise volvulus around SMA with a markedly narrow angle between SMA and AA. Dissection of the distal duodenum revealed an abnormally positioned transverse colon, significant adhesions surrounding the blood vessels, and pronounced bleeding at the operative site. Considering the patient's condition, the procedure was converted to an open laparotomy.
15–27 November 2025	Postoperative retention of the abdominal and pelvic drainage tubes.	1. The drainage volume for each tube was documented. 2. The total daily fluid output and urine volume were recorded. 3. Blood electrolyte and protein levels were monitored. 4. Intravenous nutritional support was provided.
20 November 2025	Autonomous passage of flatus.	1. The patients began engaging in suitable activities. 2. Gastrointestinal recovery was monitored.
21 November 2025	Autonomous bowel movements.	1. Limited drinking water was administered. 2. Intermittent clamping of the gastrointestinal drainage tube was implemented. 3. Gastrointestinal recovery was monitored.
23 November 2025	Initiation of a liquid diet.	1. Intermittent clamping of the gastrointestinal drainage tube was continued. 2. Gastrointestinal recovery was monitored.
30 November 2025	Gradual resumption of a regular diet, restoration of normal gastrointestinal function, and a stable condition.	The patient was discharged.

## 3 Discussion

During the embryonic period, the small intestine, ascending colon, and transverse colon primarily originate from the midgut. By the 10th week of embryonic development, the volume of the abdominal cavity increases rapidly, allowing the previously herniated midgut to return to the abdominal cavity. This process is characterized by the rotation of the midgut, led by the jejunum, around the axis of the SMA. Abnormal fixation or incomplete rotation can result in intestinal malformations and variations in anatomical positioning, a condition known as IM (4, 8). This condition can manifest in all age groups. Research indicates that the incidence of malrotation is estimated at 1:6,000–1:2,500 live births in the first year of life. Of these cases, 75%–90% are diagnosed within the first year due to the presence of characteristic symptoms. Conversely, individuals over the age of 1 year often remain undiagnosed or present with atypical symptoms, rendering the precise incidence in this age group unknown (2, 3, 9–11). In cases of abnormal band compression in the IM, there is an elevated risk of intestinal volvulus or even intestinal necrosis, which can potentially result in mortality. The hallmark symptoms of IM are bilious vomiting, abdominal pain, and weight loss (1, 2). In this case, the patient presented with acute abdominal pain and vomiting, which are consistent with the typical clinical manifestations of IM. Diagnostic approaches for IM include upper gastrointestinal series (UGIs), US, and CT examination. Although UGIs is considered the gold standard for diagnosing IM, its use is limited in the presence of intestinal obstruction (12). Consequently, US and CT imaging are preferred as first-line diagnostic tools for IM, with the “vortex sign” being a characteristic imaging feature (13, 14). In this case, US and CT imaging revealed the “vortex sign,” facilitating a definitive preoperative diagnosis of IM.

Ladd's procedure is a well-established surgical intervention, considered the definitive treatment for IM (15, 16). Traditionally, open Ladd's procedures have been regarded as the gold standard for managing IM. However, since the advent of laparoscopic techniques, laparoscopic Ladd's procedures have been successfully performed for IM treatment since 1995 (17). The clinical utilization of this minimally invasive approach has progressively expanded; however, its efficacy remains a subject of debate. Current research suggests that laparoscopic Ladd's procedures may offer certain advantages, such as shorter hospitalization and reduced postoperative pain, compared with the conventional open approach. Nevertheless, this laparoscopic technique may be associated with an increased risk of postoperative volvulus recurrence and a higher likelihood of intraoperative conversion to open surgery (1, 18–20). In this study, the case was identified as an RIM during laparoscopic exploration. Upon separation of the distal duodenum, the transverse colon was found to be abnormally positioned, with severe adhesions surrounding the mesenteric vessels and significant intraoperative bleeding. Continuing with the laparoscopic procedure would have considerably extended the operative duration. Given the patient's critical condition and limited tolerance for prolonged surgery, a decision was made to convert to a laparotomy to complete the necessary surgical intervention. Due to the unique nature of this case, modifications were made to the classical Ladd's procedure to tailor it to the patient's specific anatomical and pathological findings. In most patients with RIM, intestinal obstruction can be alleviated following primary Ladd's procedure, which involves the release of Ladd's bands, lysis of adhesions, and reduction of intestinal volvulus (21, 22). In cases where complications such as internal hernia are present, obstruction can be fully resolved by supplementing Ladd's procedure with internal hernia reduction and further surgical interventions (23). However, in the case presented here, after performing the standard Ladd's procedure, it was observed that the obstruction caused by SMA compression of the duodenum persisted, and conventional release techniques were ineffective in relieving the vascular compression. Consequently, we performed duodenojejunostomy (side-to-side anastomosis) to bypass the site of SMA compression and restore intestinal continuity. This approach represents an individualized modification of the traditional Ladd's procedure. Postoperatively, the patient demonstrated satisfactory recovery, with no procedure-related complications, restoration of gastrointestinal function, and gradual weight gain.

The prevalence of SMAS, also referred to as Wilkie’s syndrome (24), ranges from 0.1% to 0.3% (5). SMAS is characterized by a disruption of the anatomical relationship among the SMA, AA, and duodenum, resulting in a reduced angle between the SMA and AA (<22°) and a decreased distance between the duodenum and adjacent vessels (2–8 mm). Symptoms of intestinal obstruction arising from compression of the horizontal segment of the duodenum are more frequently observed in adolescents and young women (24, 25). The clinical manifestations of SMAS include abdominal pain, nausea, vomiting, weight loss, and other features indicative of chronic gastrointestinal obstruction, although they are not specific (26). The etiology of SMAS encompasses both congenital factors, such as improper intestinal rotation and a short ligament of Treitz, and acquired factors, including surgery, trauma, and tumors (27, 28). Diagnosis relies on clinical presentation in conjunction with imaging studies, with the angle between AA and SMA and aortomesenteric distance serving as critical imaging criteria. Currently, there is no standardized treatment protocol for SMAS, which includes both conservative and surgical approaches. Conservative management is appropriate for patients with mild symptoms and short disease duration and involves addressing the underlying conditions, modifying the body position, providing nutritional supplementation, and implementing smaller, more frequent meals (26). However, research indicates that 50%–70% of patients receiving conservative treatment experience symptom recurrence, necessitating surgical intervention (29). The objective of surgical management is to bypass or relieve the obstruction (30). In this case study, preoperative imaging revealed a reduced angle (10°) between the SMA and AA, accompanied by proximal duodenal dilation. Intraoperatively, the horizontal segment of the duodenum was compressed, the angle between AA and SMA was further decreased, and the SMA was positioned in close proximity to the duodenum. The patient, a girl, presented with preoperative symptoms of postural abdominal pain and vomiting, was underweight with a BMI of 16 kg/m^2^ (grade II malnutrition), and had a STAMP score of 2 (moderate risk). A clinical diagnosis of SMAS was made. She experienced an acute onset of symptoms and underwent a side-to-side duodenojejunostomy to alleviate the obstruction.

In clinical practice, the co-occurrence of RIM and SMAS is exceedingly rare. Previous reports have documented instances of abnormal vascular development due to RIM in adults, compression of the terminal ileum by SMA in cases of atypical IM, and duodenal compression, resulting in intestinal obstruction. However, these cases differ markedly from the case described here (31–33). This study highlights a rare case of RIM concomitant with SMAS. Given the complex anatomical considerations, the surgical team adapted the classical Ladd's procedure to the patient's specific anatomical configuration, resulting in favorable therapeutic outcomes.

This study has several limitations. First, the patient was admitted to the emergency department without prior consideration of SMAS; consequently, no computed tomography angiography (CTA) was performed to assess the angle between SMA and AA or mesenteric spacing, resulting in the absence of core imaging diagnostic indicators. Therefore, the diagnosis was based solely on intraoperative findings and clinical manifestations. Second, the study did not systematically assess the psychological status of the patient, and the follow-up period was relatively short. Patients experiencing long-term gastrointestinal symptoms, such as abnormal defecation, require extended follow-up for further evaluation and validation (34).

## 4 Conclusions

In patients presenting with IM and symptoms of abdominal pain and vomiting that vary with body position, clinicians should consider the potential coexistence of RIM and SMAS. When the patient's condition allows, comprehensive preoperative assessments, including CTA, should be performed to evaluate further gastrointestinal malformations. Such a thorough evaluation is essential for selecting the most appropriate surgical intervention, with the objectives of minimizing intestinal trauma, reducing operative time, decreasing intraoperative blood loss, and facilitating faster postoperative recovery of gastrointestinal function.

## 5 Patient perspective

“After eating that day, I began to experience abdominal pain. Initially, I did not inform my parents, thinking I had simply eaten something unsuitable, and instead, I lay on the ground. Although the pain was somewhat alleviated, it remained significant, and I experienced vomiting. When my parents took me to the hospital, I was in so much pain that I could not speak. I felt a profound sense of fear when the doctor told me that I required immediate surgery. Despite the doctor's attempts to comfort me, I could not stop my tears. Upon awakening after the operation, I found myself with several tubes attached to my body, which made it difficult for me to move in bed. I also experienced frequent blood draws, thirst, and hunger, which contributed to my irritability. Fortunately, as the days progressed, I was able to engage in activities, drink water, eat, and have my tubes removed. When the doctor informed me that I could be discharged from the hospital, I experienced an overwhelming sense of joy. Since then, I have learned the importance of promptly communicating any discomfort to my parents. Although the surgery was somewhat frightening, I found the doctor to be very compassionate, and I realized that I needed to be braver than I initially believed.”

## Data Availability

The raw data supporting the conclusions of this article will be made available by the authors, without undue reservation.
